# Gamified clinical case video game in occupational therapy

**DOI:** 10.1186/s12909-026-09301-9

**Published:** 2026-04-27

**Authors:** Julia Dugnol-Menéndez, Estíbaliz Jiménez-Arberas, David Fernández-Valera, Jesús Merayo-Lloves

**Affiliations:** 1 Padre Ossó Faculty (University of Oviedo), Prado Picón, S/N, Oviedo, Asturias 33008 Spain; 2 Faculty of Medicine and Health Sciences (University of Oviedo), Julián Clavería, 6, Oviedo, Asturias 33006 Spain

**Keywords:** Occupational therapy, Education, Video game, Gamification

## Abstract

**Background:**

Gamification in the classroom is gaining traction in universities and is driving educational innovation. In this context, a learning experience based on an educational video game centred on clinical cases was proposed. This study is based on education related to occupational therapy and was implemented in two interrelated courses of the program. The initiative aimed to increase student motivation through the use of an interactive and practical approach, as well as to enhance student learning.

**Methods:**

A quasi-experimental pre–post study was conducted over three academic years with 120 occupational therapy students enrolled in two related courses. A video game prototype based on clinical case resolution was developed, in which players progress by selecting correct answers and making decisions after viewing feedback videos. The game, available in Spanish and English, records scores and allows simultaneous online participation. Student performance was assessed through pre- and post-tests, and satisfaction was evaluated using a survey. Descriptive and inferential statistical analyses were performed using SPSS version 24 and Microsoft Excel.

**Results:**

Significant differences were observed in theoretical and practical knowledge before and after the game, with an overall improvement of 21.84% in test scores. Greater gains were observed in the FAI-D subject compared to FAI-MP. Very few students chose to play the English version. The video game was positively evaluated in terms of interest and usefulness for learning, especially in anatomy, where it had a notable effect on knowledge improvement.

**Conclusions:**

The gamified clinical case video game appears to be a useful complementary educational tool associated with improved learning outcomes and student motivation in occupational therapy education.

**Supplementary Information:**

The online version contains supplementary material available at 10.1186/s12909-026-09301-9.

## Introduction

During the twentieth century, a new concept of education emerged, emphasizing the importance of games in learning and cognitive development, a perspective that remains relevant today due to the impact of technological advances on education [1]. This has fostered the development of learning approaches such as blended learning and distance education [2]. There has been a growing trend in using gamification—the use of game elements and techniques in non-game contexts, such as education—as an effective strategy to create engaging learning experiences in the educational field [3]. In higher education, it has been shown to enhance motivation, participation, knowledge retention, and skill development [3, 4].

Digital games have been used for educational purposes since the 1970 s, incorporating elements such as reward systems, narrative structures, and interactive feedback that support learning [5, 6]. These characteristics have contributed to the widespread use of digital games in higher education as tools to promote active learning and skills development [7, 8]. In health sciences, their application has expanded notably, including the use of medical simulations to train clinical skills and decision-making [9], as well as their implementation in rehabilitation and health education [10, 11]. Additionally, video games and virtual reality have demonstrated benefits in motor and cognitive rehabilitation, as well as in improving patient motivation and treatment adherence [12–18].

Despite this evidence, the integration of these approaches into occupational therapy education remains limited. Occupational therapists require strong clinical reasoning skills to assess patients and adapt interventions [19], yet students often face difficulties in applying theoretical knowledge to clinical decision-making, particularly during the transition to professional practice [20, 21]. Clinical cases provide an effective strategy to address this issue by promoting analytical thinking and evidence-based decision-making in realistic scenarios [22, 23].

At the same time, the growing importance of digital competencies in healthcare highlights the need for better integration of technology into occupational therapy curricula. Although its relevance is widely recognized, students do not always achieve adequate levels of digital literacy [8, 24–30] and effective use of technology requires advanced knowledge of digital tools and evidence-based resources [30]. This underscores the need to incorporate interactive and simulation-based tools that support both professional and technological skill development [24, 25].

In this context fully developed educational video games (rather than the isolated application of gamification elements) offer a promising approach by combining clinical case resolution, interactive feedback, and autonomous learning. However, there is still a lack of educational tools specifically designed for occupational therapy that integrate clinical reasoning training with digital skill development through interactive environments.

Therefore, this study presents an interactive educational video game prototype based on clinical case resolution. The aim is to support the development of key competencies in occupational therapy, including clinical reasoning, decision-making, autonomous learning, and second-language proficiency [31, 32], through progressive challenges and real-time feedback.

### Study aims

The aims of this study were twofold:

- First, to design and implement a gamified educational video game based on clinical cases in occupational therapy education, with the aim of enhancing student motivation and engagement.
- Second, to evaluate its impact on students’ knowledge acquisition and satisfaction, and to explore its potential to promote the use of English as a second language as a transversal competence in occupational therapy training.

## Materials and methods

### Study design

A quasi-experimental pre–post study was conducted over three consecutive academic years in an occupational therapy degree program.

### Ethics statement

Owing to its educational purpose, the project was reviewed and approved by the Research Ethics Committee of the Principality of Asturias (T.F.G. nº 2020.038). Participation was voluntary, following the signing of an informed consent form where students were briefed on the study’s nature. Furthermore, data analysis was conducted anonymously.

### Participants and sample selection

The video game project “SOS! We need an… OT^©^” was trialled over the course of three consecutive years in an occupational therapy degree, specifically in the subjects of “Functional Autonomy and Independence in Musculoskeletal Pathologies” (FAI-MP) and “Functional Autonomy and Independence in Disabilities” (FAI-D), corresponding to the first and second semesters, respectively. Participants were included in the study if they completed the video game and responded to both the pre-test and post-test questionnaires. A total participation rate of 73.17% was obtained (see Table 1).

**Table 1:** Overview of total participation in the game and in each subject over the three years

		ACADEMIC YEAR			
		20–21	21–22	22–23	TOTAL
FAI-MP	Total students	35	30	39	104
	Participants	25	25	28	78
	Participation percentage	71.43%	83.33%	71.79%	75.00%
	Playing in English language	9	1	0	10
	Playing in English language percentage	36.00%	4.00%	0.00%	12.82%
	Survey satisfaction responses	16	21	28	65
	Percentage of surveys answered	64.00%	84.00%	100.00%	83.33%
		20–21	21–22	22–23	TOTAL
FAI-D	Total students	19	25	16	60
	Participants	17	16	9	42
	Participation percentage	89.47%	64.00%	56.25%	70.00%
	Playing in English language	3	0	0	3
	Playing in English language percentage	17.65%	0.00%	0.00%	7.14%
	Survey satisfaction responses	16	12	9	37
	Percentage of surveys answered	94.12%	75.00%	100.00%	88.09%
		20–21	21–22	22–23	TOTAL
FAI-MP + FAI-D	Total students	54	55	55	164
	Participants	42	41	37	120
	Participation percentage	77.78%	74.55%	67.27%	73.17%
	Playing in English language	12	1	0	13
	Playing in English language percentage	28.57%	2.44%	0.00%	10.83%
	Survey satisfaction responses	32	33	37	102
	Percentage of surveys answered	59.26%	60.00%	67.27%	85.00%

### Assessment instruments

#### Design and development of the knowledge test

A pre- and post-test was developed to assess students’ understanding of the theoretical and practical content addressed in each subject (see Additional files 1 and 2). The items were designed based on the learning objectives of the courses and the official syllabus content, including concepts directly addressed in the video game. These tests were uploaded to the university’s Moodle platform.

The same questionnaire was administered before and after the intervention to evaluate changes in knowledge after gameplay. No formal pilot testing or psychometric validation of the instrument was conducted, which should be considered when interpreting the results.

#### Design and development of the feedback survey

After playing, the students completed a satisfaction survey through Microsoft Forms, consisting of three sections (see Additional file 1): I) sociodemographic data, II) evaluation of the game categories, and III) assessment of the gamification activity. A Likert-type scale was used for both the sections related to the evaluation of game categories and the gamification activity. The students provided ratings by selecting from a graded and ordered scale, with 1 being very low and 5 being very high. Additionally, a section was included where participants could share their opinions on what they liked the most, what they liked the least, and any possible suggestions, aiming to gather more information about the methodology and learning environment. The survey was voluntary and anonymous to ensure objective results.

### Intervention: video game design

The video game was developed as an interactive educational tool based on clinical case resolution and implemented using standard web-based technologies. Its design and development were guided by the Occupational Therapy Practice Framework (4th edition), established by the American Occupational Therapy Association, which constitutes a shared conceptual framework for both students and professionals in occupational therapy.

All instructional content, including both intervention and assessment components, was grounded in principles of evidence-based occupational therapy, ensuring alignment with current scientific knowledge and best clinical practices. Furthermore, the clinical cases were structured according to discipline-specific clinical reasoning processes, reflecting authentic occupational therapy practice. This approach aimed to facilitate the acquisition of the programme’s intended learning outcomes, enabling students to integrate theoretical knowledge with professional competencies in a meaningful and applied context.

**Fig. 1:**
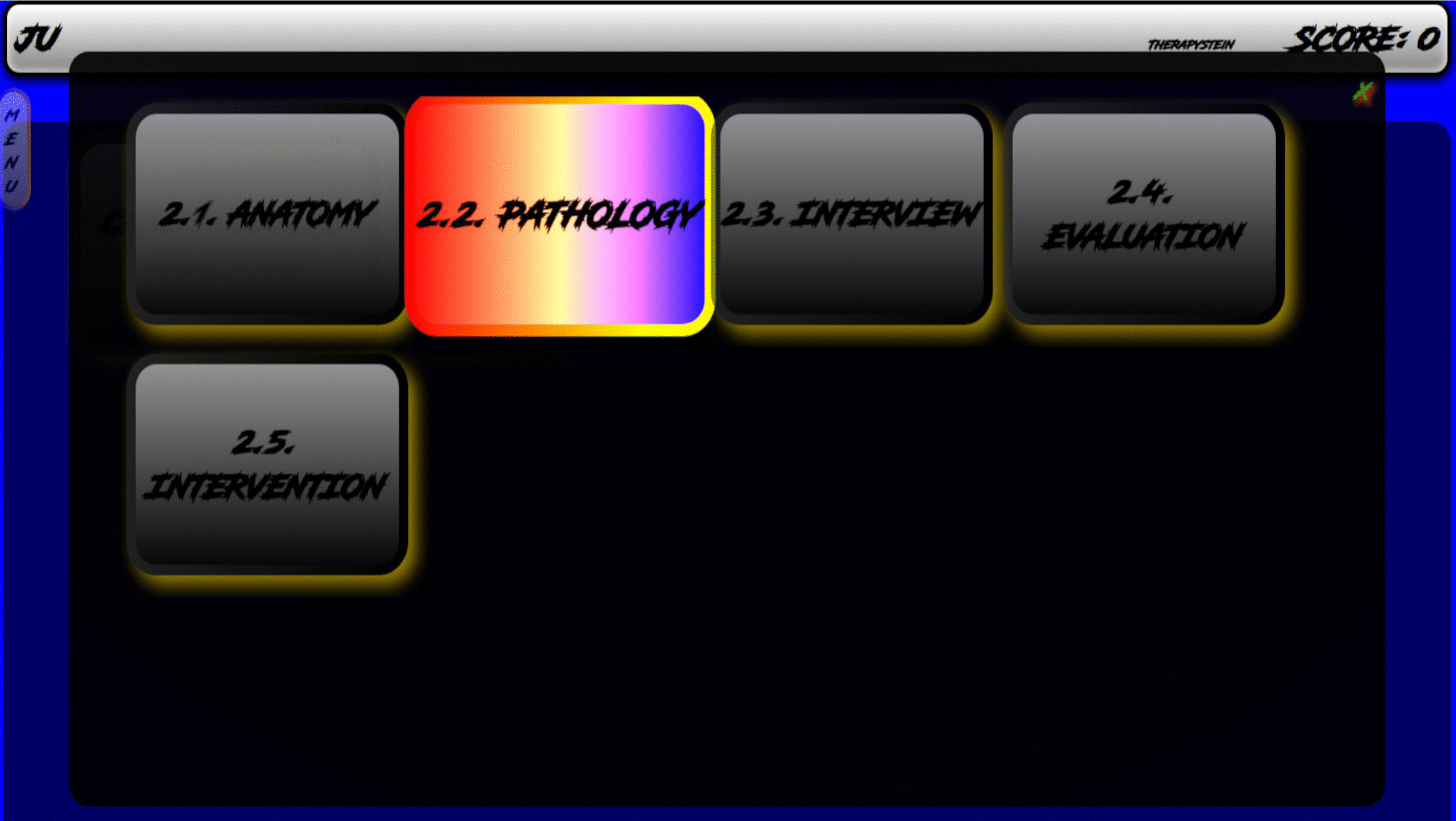
Video game screen for selecting the category within the clinical case

In addition, custom graphics, animations, and video content were specifically created for this project to enhance engagement and support the learning experience, using widely accessible digital tools to ensure scalability and usability across educational settings. The prototype of the video game is structured around a series of clinical cases, which, in turn, are divided into different categories: anatomy, pathology, interview, evaluation, and occupation (Fig. 1). Students play with the main avatar and must use their knowledge to respond correctly to the questions posed, make decisions, and progress through the case.

**Fig. 2:**
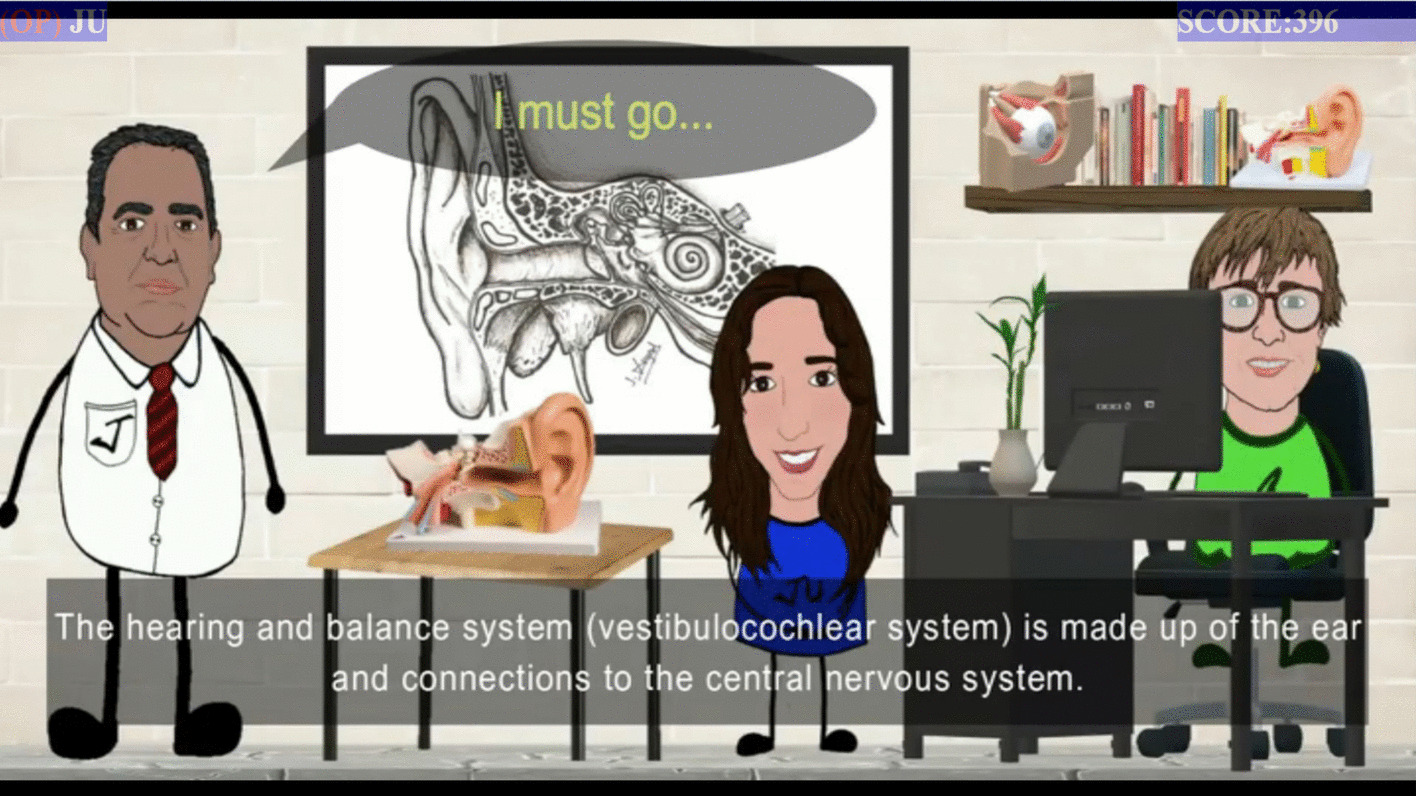
Screenshot of a fragment of the video game

**Fig. 3:**
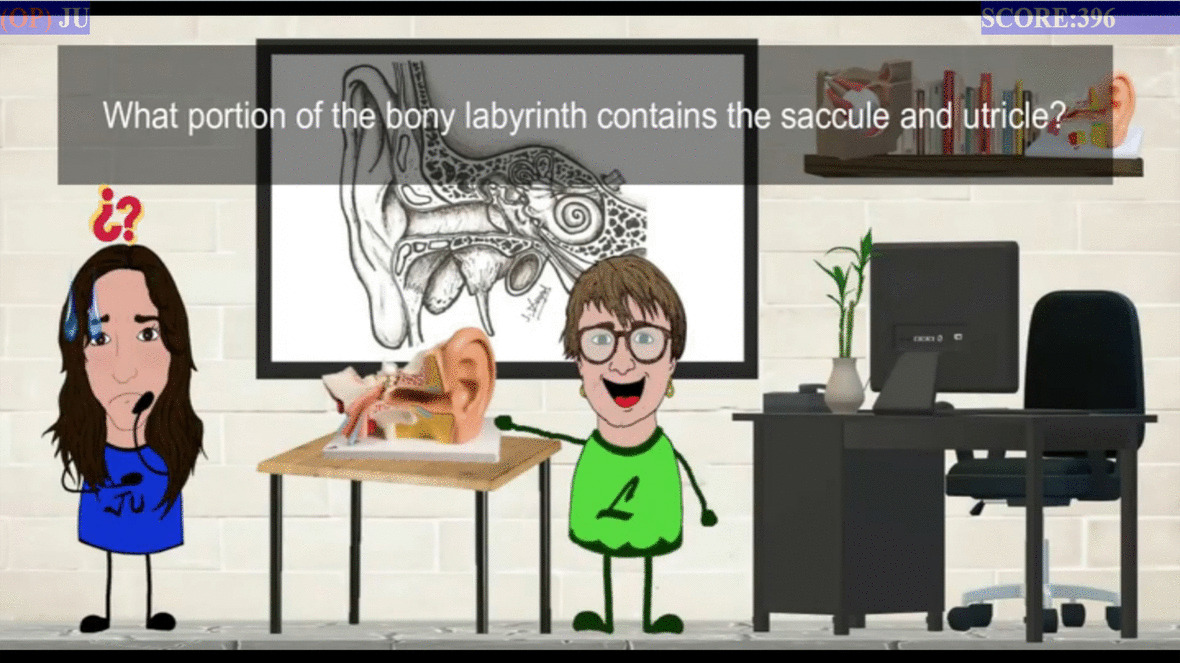
Screenshot of a game question

**Fig. 4:**
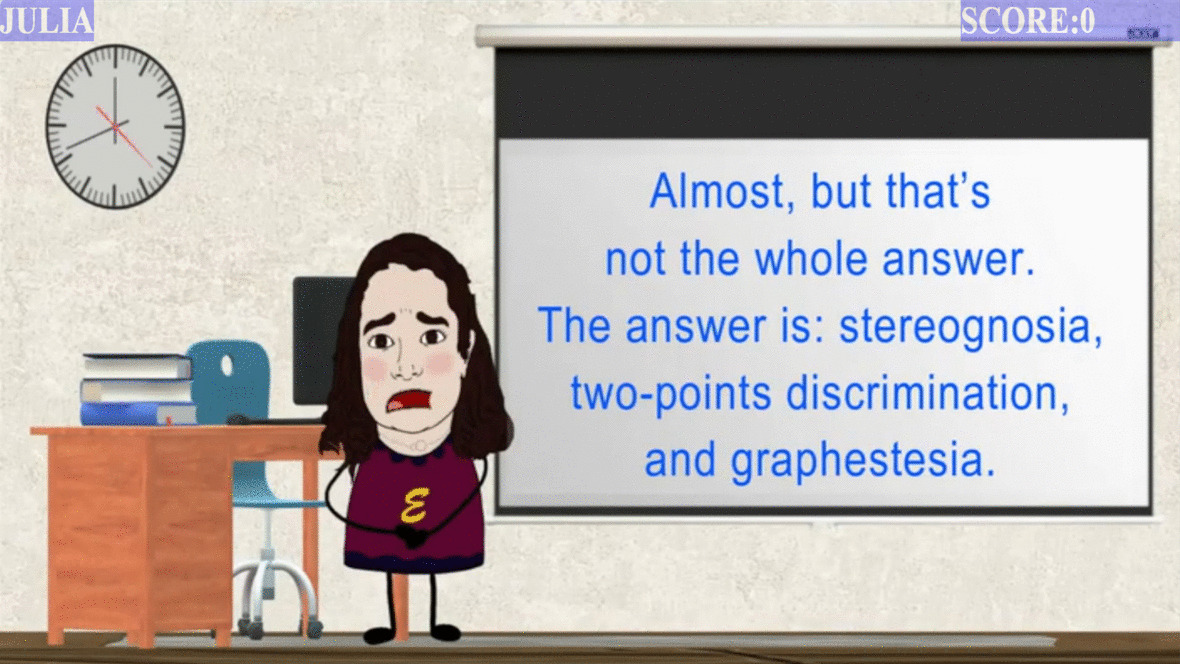
Screenshot of the feedback response to one of the questions posed

All categories begin with an introductory video with sound in either English or Spanish, depending on the selection. It is the only video without subtitles and with dialogue audio; the rest have subtitles and are silent (Fig. 2). The videos in each category are linked, and at the end of each video, a question is posed with five possible answer options (Fig. 3). At the bottom of the screen, a rapidly decreasing red-colored time bar is displayed; in the event of correctly answering the question before it disappears, an additional score is earned for response speed. An explanatory video is always provided with each answer, detailing the reasoning behind the correct choice (Fig. 4). In addition, security measures were implemented in the video game to prevent cheating since the system can detect the number of open sessions and views of the same video.

To determine the winner, the score achieved in the game was calculated according to the following aspects: 1) if all categories of the clinical case were completed with the highest number of correct answers; 2) if the students played in English; and 3) the speed of response to the questions.

### Data analysis

All data was coded and entered into the Statistical Package for the Social Sciences (SPSS version 24, Softonic S.L., Barcelona, Spain) and Microsoft Excel (Office 365 ProPlus) in preparation for statistical analysis and to generate the figures.

#### Knowledge test analysis

The scores were compared before and after the video game was played for each subject and grade, and parametric and non-parametric statistics were used to determine differences in test scores, grades, and subjects. Additionally, the results were compared between the languages and the analysed cases. Finally, the mean scores obtained for each subject and each year were compared.

#### Survey analysis

Consistent with the data analysis processes used in section I, the responses were summarised as percentages to present demographic information. For sections II and III, mode calculations, mean scores, and standard deviations were performed to evaluate the different categories of the game and the gamification activity.

## Video game results and practical application to improve learning

### Game score

The number of correct responses per student was considered for the calculation of the game score, and this information was obtained from the video game database. The speed of completion bonus and language used were not assessed. The mean game score across the three academic years was 5.21 ± 1.32 out of 10 points. Significant differences were observed between clinical cases, with higher scores in FAI-MP compared to FAI-D (*p* = 0.001). No significant differences were found between academic years (*p* = 0.210) or between students who played in English and those who played in Spanish (*p* = 0.141).

### Practical application of the interactive video game to learning memory

To assess the impact of the video game on student learning, the results of the pre- and post-video game questionnaires were compared (see Table 2). Additionally, 95% confidence intervals were calculated for the mean scores to provide an estimate of the precision of the observed differences.

**Table 2:** Differences between the pre-test and post-test scores, considering the academic year and the study case (FAI-MP/FAI-D)

Score	Academic year	Clinical case	n	M	SD	SE	Coefficient of variation	95% CI
Pre-test	20–21	FAI-MP	25	6.973	1.508	0.302	0.216	6.350–7.596
		FAI-D	17	4.941	1.391	0.337	0.281	4.227–5.655
	21–22	FAI-MP	25	6.400	1.187	0.237	0.185	5.911–6.889
		FAI-D	16	4.867	1.408	0.352	0.289	4.117–5.617
	22–23	FAI-MP	28	6.646	1.450	0.274	0.218	6.084–7.208
		FAI-D	9	5.333	1.414	0.471	0.265	4.247–6.419
Post-test	20–21	FAI-MP	25	7.953	0.964	0.193	0.121	7.555–8.351
		FAI-D	17	8.529	1.007	0.244	0.118	8.012–9.046
	21–22	FAI-MP	25	7.803	1.399	0.280	0.179	7.225–8.381
		FAI-D	16	8.600	1.020	0.255	0.119	8.056–9.144
	22–23	FAI-MP	28	7.621	1.065	0.201	0.140	7.209–8.033
		FAI-D	9	7.778	1.481	0.494	0.190	6.639–8.917

*M* Mean, *SD* Standard deviation, *SE* Standard error, *95% CI* 95% confidence interval of the mean

Overall, improvements were observed across all academic years and clinical cases. Considering the three academic years and both clinical cases, an overall improvement of 21.84% was obtained (M ± SD (before) = 6.09 ± 1.6; M ± SD (after) = 7.79 ± 1.18). Greater improvements were observed in FAI-D (40.48%) compared to FAI-MP (14.31%). The lowest overall performance was observed in the 2022–2023 academic year (see Table 3).

**Table 3:** Percentage of improvement by academic year and clinical case

Academic year	FAI-MP	FAI-D	GLOBAL
20–21	12.32%	42.07%	24.86%
21–22	17.98%	43.41%	28.05%
22–23	12.80%	31.43%	17.40%
Total	14.31%	40.48%	21.84%

To determine whether these differences were statistically significant, a repeated measures analysis of variance (ANOVA) was conducted comparing pre-test and post-test scores across academic years and clinical cases.

Assumptions of normality and homogeneity of variance were examined before conducting the repeated measures ANOVA (Table 4).

**Table 4:** Shapiro–Wilk normality test by academic year

	**Academic year**	**Significance**	**gl**	**LoSig**
Pre-test	20–21	0.974	42	0.439
	21–22	0.964	41	0.209
	22–23	0.957	37	0.164
Post-test	20–21	0.952	42	0.078
	21–22	0.935	41	0.021
	22–23	0.971	37	0.439

The analysis revealed statistically significant differences between pre-test and post-test scores overall (F (1, 114) = 300.005, *p* < 0.001), indicating an overall improvement after the intervention. Significant differences were also observed according to academic year (F (2, 114) = 3.617, *p* = 0.030) and clinical case type (F (1, 114) = 71.503, *p* < 0.001).

An examination of the differences between courses and clinical cases revealed that improvement was not influenced by an interaction between case and academic year (F (2, 114) = 1.664, *p* = 0.194). No significant differences were observed between academic years (F (2, 114) = 0.498, *p* = 0.609), indicating that no course performed better than others. However, significant differences were found between clinical cases (F (1, 114) = 6.618, *p* = 0.011).

**Fig. 5:**
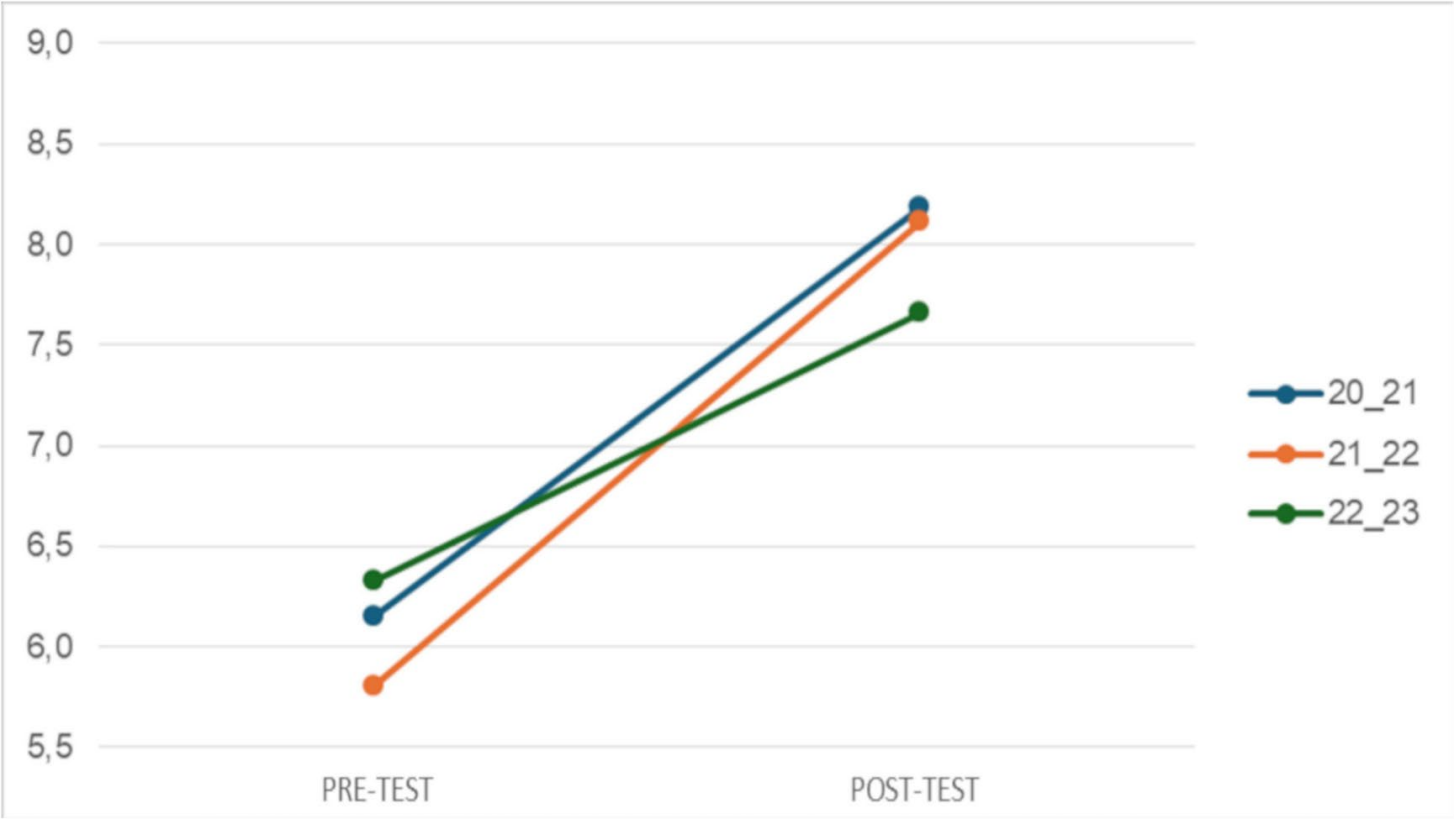
Differences between the pre-test and the post-test throughout the academic years, considering both clinical cases and languages

Furthermore, no significant differences were observed between academic years (F (2, 114) = 0.498, *p* = 0.609), whereas differences between clinical cases remained significant (F (1, 114) = 6.618, *p* = 0.011). This difference may be related to the fact that FAI-MP is associated with the second year of the occupational therapy program, whereas FAI-D corresponds to the fourth year (see Table 5 and Fig. 5).

**Table 5:** Inter-subject effects based on academic year and clinical case (FAI-MP and FAI-D)

Variable	SS	Gl	MS	F	p	ŋ^2^
Academic year	2.423	2	1.211	0.498	0.609	0.003
Clinical case	16.088	1	16.088	6.618	0.011	0.023
Academic year*clinical case	1.294	2	0.647	0.266	0.767	0.002
Residuals	277.145	114	2.431			

Sum of squares type III

*SS* Sum of squares, *MS* Mean square

**Fig. 6:**
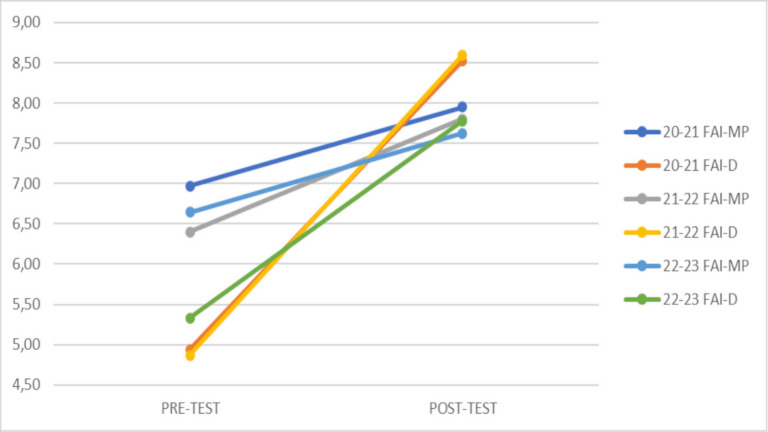
Comparison by academic year and clinical case developed

Differences between pre-test and post-test scores across academic years and clinical cases indicated a clear distinction between FAI-MP and FAI-D. Overall, higher scores were observed in post-tests across all academic years, with FAI-D showing consistently higher scores (Table 2; Fig. 6).

Post-hoc comparisons were performed to identify differences between academic years and clinical cases in pre-test and post-test scores. The greatest improvements were observed in the 2021–2022 academic year, followed by 2020–2021 and 2022–2023 (Table 6). Significant differences were found across clinical cases: FAI-MP showed higher scores in the pre-test, whereas FAI-D showed higher scores in the post-test (Table 7 and Fig. 6).

**Table 6:** Post-hoc tests by academic year and pre-test and post-test

		**MD**	**ET**	**t**	**p**_**Holm**_
20–21, Pre-test	21–22, Pre-test	0.324	0.286	1.132	1.000
	22–23, Pre-test	−0.032	0.316	−0.102	1.000
	20–21, Post-test	−2.284	0.202	−11.315	< 0.001
	21–22, Post-test	−2.244	0.286	−7.840	< 0.001
	22–23, Post-test	−1.742	0.316	−5.511	< 0.001
21–22, Pre-test	22–23, Pre-test	−0.356	0.319	−1.119	1.000
	20–21, Post-test	−2.608	0.286	−9.111	< 0.001
	21–22, Post-test	−2.568	0.206	−12.493	< 0.001
	22–23, Post-test	−2.066	0.319	−6.487	< 0.001
22–23, Pre-test	20–21, Post-test	−2.252	0.316	−7.121	< 0.001
	21–22, Post-test	−2.212	0.319	−6.944	< 0.001
	22–23, Post-test	−1.710	0.246	−6.950	< 0.001
20–21, Post-test	21–22, Post-test	0.040	0.286	0.139	1.000
	22–23, Post-test	0.542	0.316	1.713	0.531
21–22, Post-test	22–23, Post-test	0.502	0.319	1.575	0.585

The results are averaged across the levels of clinical case

*MD* Mean difference, *ET* Standard error

**Table 7:** Post-hoc tests by clinical case and pre-test and post-test

		**MD**	**ET**	**T**	**p**_**Holm**_
AIF-PO, Pre-test	AIF-D, Pre-test	1.626	0.251	6.479	< 0.001
	AIF-PO, Post-test	−1.120	0.146	−7.688	< 0.001
	AIF-D, Post-test	−1.630	0.251	−6.494	< 0.001
AIF-D, Pre-test	AIF-PO, Post-test	−2.745	0.251	−10.940	< 0.001
	AIF-D, Post-test	−3.255	0.206	−15.773	< 0.001
AIF-PO, Post-test	AIF-D, Post-test	−0.510	0.251	−2.033	0.044

The results are averaged across the levels of academic year

*MD* Mean difference, *ET* Standard error

Regarding language, it should be noted that no participants played in English in the 2022–2023 academic year, and only one student did so in 2021–2022. Therefore, the analysis mainly reflects the 2020–2021 cohort. Significant differences between pre-test and post-test scores were observed (see Table 8). However, no substantial improvement was associated with the language used. A small effect related to overall performance was identified (η^2^ = 0.046), suggesting slightly higher scores irrespective of language.

**Table 8:** Inter-subject effect based on language and clinical case (FAI-MP and FAI-D)

Score	SS	gl	MS	F	p	ŋ^2^
Overall score	68.948	1	68.948	87.268	< 0.001	0.314
Score*language	0.635	1	0.635	0.804	0.376	
Score*clinical case	26.586	1	26.586	33.650	< 0.001	0.121
Score*language*clinical case	0.049	1	0.049	1.201	1.201	
Residuals	30.023	38	0.790			

Type III Sum of Squares; η^2^ = Effect Size (considered only for significant results)

*SS* Sum of squares, *MS* Mean square

### Feedback survey results

Over three consecutive academic years, 120 students from the occupational therapy degree program participated, representing 72.00% of the total cohort. Of these, 102 students completed the satisfaction survey (85.00% of participants), including 21 male (20.59%) and 81 female (79.41%) students. Participants’ ages ranged from 18 to 30 years, with most aged 18–25 years.

The results obtained from the “evaluation of the game categories” section demonstrate a good level of satisfaction. Scores consistently exceeded 3.5 out of 5 points in all aspects related to the evaluated parameters (see Table 9). The category with the lowest rating was anatomy, possibly due to students’ challenges in recalling knowledge from previous years, as highlighted in open-ended responses under “weaknesses” and “areas for improvement”.

**Table 9:** Satisfaction survey results

**Item:** Evaluation of the game categories (1 Very Low – 5 Very High)	**M (*SD*)**	**Mo**	**N (%)**
1) Anatomy	3.8 (0.83)	4	58 (56.90)
2) Pathology	3.87 (0.88)	4	52 (51.00)
3) Interview	4.29 (0.68)	4	52 (51.00)
4) Evaluation	4.29 (0.75)	4	29 (28.40)
5) Intervention	4.41 (0.64)	5	31 (30.40)
6) Occupation	4.3 (0.73)	4	48 (47.10)

**Item:** Evaluation of Gamification Activity (1 Very Low – 5 Very High)	**M (*SD*)**	**Mo**	**N (%)**
1) Level of interest in the activity	4.23 (0.53)	4	69 (67.60)
2) Level of utility for learning	4.42 (0.68)	5	51 (50.00)
3) Level of utility for reinforcing prior knowledge	4.45 (0.54)	4	52 (51.00)
4) Theoretical knowledge provided by the teachers for the development of the case	4.32 (0.68)	4	48 (47.10)
5) Communication and willingness on the part of the teachers for resolving doubts	4.4 (0.71)	5	53 (52.00)

*M* Mean, *SD* Standard deviation, *Mo* Mode, *N* Frequency, *%* percentage

The evaluation of the gamification activity revealed excellent satisfaction levels, with scores consistently exceeding 4 out of 5 points across all assessed parameters.

The data obtained from the open-ended question, “strengths”, can be grouped into the following categories:

1. Interactivity and dynamism: Students highlighted the game’s interactivity and how it aided the understanding of concepts through animations and detailed explanations in case of mistakes. They also appreciated the integration of different subjects and how they related to each other.
2. Utility and knowledge reinforcement: Participants emphasized the game’s utility for reinforcing, applying, and internalizing knowledge acquired previously during classes and practices, especially in anatomy.
3. Realism and practical applicability: The realistic approach of the game to occupational therapy situations was positively valued, with scenarios reflecting professional practice.
4. Clarity and detailed explanations: The detailed explanations provided in the game were praised, especially when errors were made, as they helped to better understand concepts and resolve doubts.
5. Variety of content and themes: Students found the variety of topics covered in the game interesting and the relevance of the questions posed, from anatomy and pathology to interview and occupation in complete clinical cases.
6. Originality and creativity: The game’s originality and creativity were highlighted as different and more interesting ways of learning, emphasizing the fun provided by the game’s playful approach. Additionally, it was more immersive, as it reminded them of their real teachers through the characters.

The data obtained from the open-ended question, “weaknesses”, can be grouped into the following categories:

1. Time limitations: It was repeatedly mentioned that the allotted time to read and answer questions was insufficient, leading to mistakes and difficulties in understanding response options. However, this was due to misinterpretation of the instructions, as the expired time bar did not result in any penalties.
2. Visualization and accessibility challenges: Some difficulty was indicated with the visualization of questions and answers, especially when the background hindered readability. Furthermore, participants often forgot the question content when selecting the response, as it disappeared when the response options were displayed.
3. Specific content and complexity: Some students found certain questions too specific or complex, particularly in the anatomy and pathology sections. Notably, long responses further hindered understanding and decision-making within the allotted time.
4. Technical and operational aspects: Technical issues, such as game slowness at certain points, lack of dialogue repetition, and inability to review questions once response options were displayed, were observed.

To conclude, the data obtained from the open-ended question, “areas for improvement”, can be grouped into the following categories:

1. User experience improvements: Suggestions were made to improve the contrast between the background and text of the responses for better readability.
2. Expansion and diversification of content: Adding more sections or pathologies to increase topic variety was suggested. Additionally, to balance game content, the possibility of including more questions on evaluation and intervention and fewer questions on anatomy was highlighted.
3. Interface and question presentation adjustments: There were suggestions to keep the question statement visible while selecting responses. The need for more time to choose responses and the possibility of rereading questions to ensure adequate understanding was noted. Simplifying the presentation of response options to save player time was also proposed, so they would not have to hover over the option to read the response.
4. Technical optimization and operational improvements: Technical issues, such as game slowness at certain points and lack of dialogue repetition, were mentioned and could be addressed to improve the overall experience. Finally, earlier notification of the date on which the game could be played to allow better preparation of theoretical content was suggested.

Despite the identified areas for improvement, overall satisfaction with the gamification activity was high among participants, who valued its educational effectiveness and interactive nature.

## Discussion

Scientific literature has already reported the effectiveness of learning games as the most positive teaching method for students [33]. Additionally, anatomy education has had to adapt to the integration of new teaching modalities, with the incorporation of new technologies shown to increase interest and knowledge retention [34]. Khaldi et al. [35] conducted a systematic literature review to identify game elements and theories used in empirical studies and proposed approaches for gamifying e-learning systems in higher education. They found that point systems, badges, and leaderboards are the most frequently used elements for gamifying e-learning systems but observed an increasing use of deeper elements such as challenges and narratives. Paradoxically, badges, leaderboards, competitions, and points are the game design elements most frequently cited as causing negative effects, including a lack of impact, worsened performance, motivational problems, lack of understanding, irrelevance, and ethical issues related to cheating in the game [36]. During the game, it was observed that certain participants had two windows open: one to respond correctly after watching the feedback video, to obtain the quick response bonus, or to try to change one answer for another once it was sent. These participants were automatically disqualified.

One aim of the video game was to encourage the use of English among the students through the English version of the game, since proficiency in a second language is one of the intersectional competencies in clinical practice outlined in the white paper for the bachelor’s degree in occupational therapy [31]. These competencies and learning outcomes, such as the use of a second language or technologies, are perceived as aspects needing improvement in their training curriculum [32]. This fact was evident because only 10.83% (*n* = 13) of the students played in English. When analysing the effects of the game between subjects, some differences were observed between groups irrespective of improvement in the pre- and post-tests. However, due to the small number of participants who used the English version, these findings should be interpreted with caution. Rather than drawing firm conclusions, these results may indicate a descriptive trend that warrants further investigation in future studies. No clear evidence was found that the percentage of improvement differed depending on the language used.

It is also noteworthy to mention the recorded level of interest and usefulness of the video game for learning—the scores listed were 4.23 and 4.42 out of five points, respectively. Motivation among students has declined in recent years, and the use of video games may be a suitable strategy among university students, as supported by literature indicating that visual learning enhances knowledge acquisition [37]. In fact, visualization is integral to learning anatomy, and demonstrating the functional importance of anatomical structures in a clinical context facilitates memorization and knowledge transfer [38]. As highlighted by the strengths of the satisfaction survey, this usefulness was particularly notable in the field of anatomy, a subject often perceived as challenging by students with concerns about acquiring anatomical knowledge [39], so gamification appears to be a useful tool to address this issue. For example, this game category received a satisfaction rating of 3.8 out of 5 points, with the majority rating it at 4 points (56.90%), indicating a fairly high level of satisfaction. In fact, students often express greater satisfaction, engagement, and motivation with gamified systems, highlighting the effectiveness of gamification in anatomy [40]. Moreover, the satisfaction survey ratings align with the results of the pre- and post-tests, where a statistically significant percentage of improvement in evaluated knowledge was observed in all cases after playing the video game, especially in the FAI-D subject. This interpretation is further supported by students’ qualitative feedback, which frequently highlighted difficulties in anatomy-related content. This alignment between quantitative and qualitative findings reinforces the interpretation of anatomy as a particularly challenging yet relevant learning area. These findings suggest that the observed improvements may be educationally relevant, as they reflect meaningful gains in students’ understanding of clinically relevant content. Overall, the interactive video game was associated with improvements in students’ performance in terms of learning memory, with differences observed both across academic years and between the different clinical cases analysed. Interestingly, in the clinical case of FAI-MP, students achieved better results in the pre-test (before playing), whereas in FAI-D, better results were obtained in the post-test (after playing). Curricula should provide continuous review of anatomical knowledge with progressive integration of clinical contexts as students advance, thereby promoting deeper learning and better application of anatomical knowledge to diverse situations [39]. In addition to statistical significance, effect sizes (η^2^) were considered to better understand the magnitude of the observed differences. The results indicated small to moderate effect sizes which suggest that, beyond statistical significance, the intervention had a meaningful educational impact on students’ learning outcomes. The relatively narrow confidence intervals observed in most groups suggest a consistent effect of the intervention across participants.

However, while our work coincides with the results of many studies supporting gamification, which indicate its positive effects on students’ intrinsic motivation and behaviour [41], a rigorous study of game effectiveness and real impact is warranted, relying on empirical evidence to clarify whether gamification truly works and under which conditions [7]. It is important to note that gamification must be carefully planned and executed to be effective and not detract from the learning objective [42]. Indeed, after reviewing several empirical studies on gamification, Hamari et al. [8] reported that while most quantitative studies concluded positive effects of gamification, these effects were evident only in some of the relationships between gamification elements and the studied outcomes. Furthermore, studies that qualitatively analysed gamification discovered that this phenomenon is very complex, with underlying factors that can confound the results, such as the contextual role being gamified and the characteristics of the users.

These findings should be interpreted with caution, as several factors may have influenced the observed results. Differences between subjects, variations in prior knowledge, and the voluntary nature of participation may have contributed to the outcomes. In particular, students with higher motivation or prior academic performance may have been more likely to participate and engage with the activity, potentially introducing selection bias. These factors should be considered when interpreting the educational impact of the intervention.

## Conclusions

The use of a gamified clinical case video game in occupational therapy education was associated with improvements in students’ knowledge and high levels of satisfaction. These findings suggest that this type of educational tool may support the development of transversal competencies, such as clinical reasoning, autonomous learning, and decision-making.

The results also indicate that the integration of clinical case—based gamification may be particularly useful for reinforcing previously acquired knowledge, especially in complex subjects such as anatomy.

From a pedagogical perspective, the development of this video game based on clinical case resolution represents a potentially valuable complementary resource for occupational therapy education. The materials, including videos, images, and assessment components, were specifically created by the authors to ensure coherence with the discipline and alignment with occupational therapy principles. This approach may support the development of both discipline-specific knowledge and professional skills, such as clinical reasoning and the application of theoretical concepts in practice. Furthermore, it offers educators a flexible and transferable tool that can be integrated into existing curricula to enhance student engagement and learning.

Future research should further explore the implementation of gamified learning strategies across different educational contexts within occupational therapy, as well as their potential role in promoting second-language use among students. In addition, further studies are needed to examine their effectiveness in supporting the acquisition of foundational knowledge and its transfer to clinical reasoning and practice. Longitudinal research would also be valuable to assess whether these learning outcomes are sustained over time.

## Study limitations

Since the activity was voluntary, the results obtained cannot be applied to all students, as they may only reflect the experience and expectations of those who participated. Furthermore, selection bias may have occurred because more motivated students may be more likely to participate.

The absence of a control group limits the ability to attribute the observed improvements exclusively to the intervention.

Moreover, the pre- and post-tests were specifically developed for this study and were not subjected to formal psychometric validation, which may limit the robustness of the measurement of knowledge gain.

Finally, the findings may have limited generalizability, as the study was conducted within a single academic program and institutional context. Future research should aim to replicate these findings in different educational settings and with more diverse student populations, as well as consider the inclusion of control groups and validated assessment instruments.

## Supplementary Information

Additional file 1. Test of FAI-MP. Description of data: the same test was used as both the pre-test and post-test to ascertain the level of understanding of the concepts related to the theoretical and practical content of FAI-MP.

Additional file 2. Test of FAI-D. Description of data: the same test was used as both the pre-test and post-test to ascertain the level of understanding of the concepts related to the theoretical and practical content of FAI-D.

Additional file 3. Satisfaction survey. Description of data: the satisfaction survey was developed to gather students’ feedback on their learning experience with the video game. The survey aims to evaluate students’ perceptions and identify areas for improvement.

## Data Availability

The datasets generated and/or analysed during the current study are not publicly available [since they contain information that could compromise the privacy of research participants] but are available from the corresponding author upon reasonable request.
