# Supplementary material for: Gamified clinical case video game in occupational therapy
Source: BMC Med Educ. 2026 Apr 27;26:949. doi: 10.1186/s12909-026-09301-9 (PMC13251296; doi:10.1186/s12909-026-09301-9)
Supplement: Supplementary file 1 — Additional file 1. Test of FAI-MP. Description of data: the same test was used as both the pre-test and post-test to ascertain the level of understanding of the concepts related to the theoretical and practical content of FAI-MP. [file 12909_2026_9301_MOESM1_ESM.pdf]

TEST CASE 1 (FAI-MP)

USERNAME IN THE GAME: ____

Please answer the following questions. Only one answer is correct. If you're unsure, feel free to skip the question:

1. **Which type of movement does not allow for the articulation of the wrist joint?**
   a) Rotation.
   b) Extension.
   c) Circumduction.
   d) Flexion.
   e) Ulnar and radial deviation.

2. **Why is the radial artery not affected by carpal tunnel syndrome?**
   a) Because it is a stronger structure than the median nerve.
   b) Because it does not pass through the carpal tunnel.
   c) Due to the pressure within the artery.
   d) Because it is a muscular artery.
   e) Options a) and d) are correct.

3. **Which muscles of the thenar eminence are affected by compression of the median nerve and would explain the atrophy of the thenar eminence?**
   a) Adductor pollicis, flexor pollicis brevis, and opponens pollicis.
   b) Adductor pollicis, abductor pollicis brevis, and opponens pollicis.
   c) Palmaris brevis, adductor pollicis, and opponens pollicis.
   d) Abductor pollicis brevis, flexor pollicis brevis, and opponens pollicis.
   e) Flexor pollicis longus, flexor pollicis brevis, and palmaris brevis.

4. **Which of the following muscles' tendons pass through the carpal tunnel?**
   a) Superficial flexor of the fingers.
   b) Long flexor of the thumb.
   c) Deep flexor of the fingers.
   d) Options a) and c) are correct.
   e) Options a), b) and c) are correct.

5. **Which evaluation tool would you use to screen if a user has depression in order to refer them for psychological services?**
   a) Yesavage test.
   b) MMSE (*Mini-Mental State Examination*).
   c) MoCa test.
   d) DASH (*The Disabilities of the Arm, Shoulder and Hand questionnaire*).
   e) ABAS-II (*Adaptive Behaviour Assessment System - Second Edition*).

6. **What is a tool for assessing occupational history?**
   a) Yesavage test.
   b) OPHI-II (*Occupational Performance History Interview – Second Edition*).
   c) DASH (*The Disabilities of the Arm, Shoulder and Hand questionnaire*).
   d) ICF (*The International Classification of Functioning, Disability and Health*).
   e) MoCa test.

7. **The plaster/fiberglass cast position in a Colles fracture should have:**
   a) 15-20º ulnar deviation.
   b) The wrist placed in supination.
   c) 15-20º radial deviation.
   d) The wrist set in dorsal flexion.
   e) Options b) and c) are correct.

8. **When we have intervened in terms of preparation methods and tasks in a Colles fracture, our intervention will likely be aimed at…**
   a) Group interventions.
   b) Advocacy.
   c) Therapeutic use of occupations and activities.
   d) Education and training.
   e) None of the options are correct.

9. **When a new user starts in the Occupational Therapy service, it is important and fundamental to…**
   a) Make a splint.
   b) Start intervening and then evaluate as needed.
   c) Establish a good therapeutic relationship.
   d) Achieve all the user's goals, even if they are not realistic at first.
   e) Impose the objectives that the user must reach at all times.

10. **Which of the following options does not occur in a Colles fracture?**
   a) Radial lengthening.
   b) Radial comminution.
   c) Dorsoradial displacement.
   d) Radial angulation.
   e) None of the options are correct.

11. **Among the complications arising from a Colles fracture, we find…**
   a) Compression syndrome.
   b) Common extensor tendon rupture.
   c) Wrist stiffness due to muscle deformity.
   d) Carpal tunnel syndrome.
   e) None of the options are correct.

12. **If you have carpal tunnel syndrome, you should avoid...**
   a) Compensatory postures with the shoulder.
   b) Performing tridigital grips.
   c) Continuing the activity for a prolonged period.
   d) Options a), b) and c) are incorrect.
   e) Options a), b) and c) are correct.

13. **According to the muscles affected by the compression of the median nerve, which of the following actions would be altered?**
   a) Extension of the fingers.
   b) Abduction of the fingers.

c) Grasp.
d) Options a) and b).
e) None of them.

14. A subterminal opposition grip…

a) Is established by opposing the thumb against the palmar surface of any of the other fingers.
b) Is used to grasp bulky objects.
c) Uses all the fingers in opposition to the palm, except the thumb.
d) Involves dynamic and varied hand movements, such as using scissors or a lighter.
e) Is established between the fingertip of the thumb and the ulnar medial side of the index finger.

15. What type of grip and movement would our user use if they had to water a bonsai with a teaspoon?

a) A centered grip along with a circumduction movement.
b) A subterminal opposition and a radial mobilization.
c) They would use a tridigital grip along with wrist flexion-extension.
d) A subterminal-lateral opposition and an ulnar mobilization.
e) They would perform a subterminal-lateral grip along with pronosupination.
