# Supplementary material for: Gamified clinical case video game in occupational therapy
Source: BMC Med Educ. 2026 Apr 27;26:949. doi: 10.1186/s12909-026-09301-9 (PMC13251296; doi:10.1186/s12909-026-09301-9)
Supplement: Supplementary file 2 — Additional file 2. Test of FAI-D. Description of data: the same test was used as both the pre-test and post-test to ascertain the level of understanding of the concepts related to the theoretical and practical content of FAI-D. [file 12909_2026_9301_MOESM2_ESM.pdf]

TEST CASE 2 (FAI-D)

USERNAME IN THE GAME: ____

Please answer the following questions. Only one answer is correct. If you're unsure, feel free to skip the question:

1. Usher syndrome type III is the result of mutations in a transmembrane protein called:

a) Clarin-1 (CLRN-1).
b) Myosin II.
c) Cadherin-3.
d) Retinin-3.
e) None of the options are correct.

2. Which of the following symptoms is associated with retinitis pigmentosa?

a) Metamorphopsia.
b) Sudden vision loss.
c) Decreased central vision.
d) Difficulty adapting to darkness.
e) Appearance of rainbow-colored halos around bright lights.

3. In the cortical sensory function that is essential in the deafblind population, we find:

a) Graphesthesia.
b) Stereognosis.
c) Two-point discrimination.
d) Proprioceptive: kinaesthesia, baresthesia, and weight discrimination.
e) Options a), b), and c) are correct.

4. The posterior chamber of the eye corresponds to the space:

a) Located posterior to the lens.
b) Located between the cornea and the sclera.
c) Located between the retina and the choroid.
d) Bound in the anterior direction by the iris and ciliary body; and bound in the posterior direction by the anterior face of the vitreous body.
e) Located between the anterior face of the iris and the posterior face of the cornea.

5. Indicate the correct option:

a) The tympanic ramp starts in the tympanic membrane.
b) The vestibular ramp starts in the vestibule.
c) The vestibulocochlear or acoustic nerve is the IX cranial nerve.
d) The cochlear dome is the base of the cochlea.
e) All options are correct.

6. The receptors of the saccule and utricle provide sensitivity in relation to:

a) Balance.
b) Vibration.
c) Hearing and linear velocity.
d) Hearing.
e) Gravity and linear acceleration.

7. **If we wanted to carry out an assessment at the motor and processing level in occupational performance, what would be the most appropriate tool for a case of deafblindness?**
   a) ALSAR (*Assessment of Living Skills and Resources*).
   b) ABAS - II (*Adaptive Behavior Assessment System – Second Edition*).
   c) PRPP (*Perceive, Recall, Plan & Perform*).
   d) AMPS (*Assessment of Motor and Process Skills*).
   e) Any of the above options.

8. **What type of intervention involves "participating in a pattern of self-care activities"?**
   a) Assistive technology.
   b) Relationships.
   c) Occupations.
   d) Activities.
   e) Preparation of methods and tasks.

9. **We want to stimulate the mechanoreceptors of a user with Usher syndrome type 3, especially those involved in texture recognition. Which ones would they be?**
   a) Ruffini corpuscles.
   b) Meissner corpuscles.
   c) Pacinian corpuscles.
   d) Krause corpuscles.
   e) Merkel nerve endings and Meissner corpuscles.

10. **What tool/model/framework of evaluation would you use to choose an assistive product or technology for the vision of a user with Usher syndrome type 3?**
    a) Method for the systematic use of technical aids.
    b) Matching Method Technology (MPT).
    c) Stimulation Activities of Daily Living (SADL).
    d) Psychosocial Impact of Assistive Devices Scale (PIADS).
    e) American Occupational Therapy Association (AOTA) Framework.
