# Supplementary material for: Gamified clinical case video game in occupational therapy
Source: BMC Med Educ. 2026 Apr 27;26:949. doi: 10.1186/s12909-026-09301-9 (PMC13251296; doi:10.1186/s12909-026-09301-9)
Supplement: Supplementary file 3 — Additional file 3. Satisfaction survey. Description of data: the satisfaction survey was developed to gather students’ feedback on their learning experience with the video game. The survey aims to evaluate students’ perceptions and identify areas for improvement. [file 12909_2026_9301_MOESM3_ESM.pdf]

VIDEO GAME SATISFACTION SURVEY

This survey is part of a teaching innovation project aimed at gathering students' opinions on the educational video game experience. Your feedback will help us improve learning, prioritize content, and choose the most effective playful tools and teaching methodologies.

We kindly request your participation in this survey. If you have any questions or need further clarification, please do not hesitate to contact us.

Thank you for your time and for contributing to this important educational research.

I give my consent to participate in the research study:

☐ YES

☐ NO

I) SOCIODEMOGRAPHIC DATA

1. Please, indicate your gender:

a) Female.
b) Male.
c) Non-binary.
d) Prefer not to disclose.

2. Please, indicate your age group:

a) 18 – 20.
b) 21 - 25.
c) 26 - 30.
d) 31 – 35.
e) 36 - 40.
f) > 40.
g) Prefer not to disclose.

II) EVALUATION OF THE GAME CATEGORIES

3. Please, rate your evaluation of each category of the game (1 being very low and 5 being very high). In case you have not been able to play all of them, indicate Not sure / No comment (NS/NC).

	1	2	3	4	5	NS/NC
**Anatomy**	☐	☐	☐	☐	☐	☐
**Pathology**	☐	☐	☐	☐	☐	☐
**Evaluation**	☐	☐	☐	☐	☐	☐
**Intervention**	☐	☐	☐	☐	☐	☐
**Occupation**	☐	☐	☐	☐	☐	☐

III) ASSESSMENT OF THE GAMIFICATION ACTIVITY

4. Please rate your evaluation of each statement (1 being very low and 5 being very high):

	**1**	**2**	**3**	**4**	**5**	**NS/NC**
**Level of interest in the activity**	☐	☐	☐	☐	☐	☐
**Level of utility for learning**	☐	☐	☐	☐	☐	☐
**Level of utility for reinforcing prior knowledge**	☐	☐	☐	☐	☐	☐
**Theoretical knowledge provided by the teachers for the development of the case**	☐	☐	☐	☐	☐	☐
**Communication and willingness on the part of the teachers for resolving doubts**	☐	☐	☐	☐	☐	☐

OPEN-ENDED QUESTIONS

5. What would you say are the strengths of the video game project?

____

____

____

____

6. Were there any particular weaknesses in the video game project?

____

____

____

____

7. Do you have any ideas on possible areas for improvement?

____

____

____

____

THANKS FOR YOUR PARTICIPATION!
